# Bio-physical characteristics of gastrointestinal mucosa of celiac patients: comparison with control subjects and effect of gluten free diet-

**DOI:** 10.1186/1471-230X-11-119

**Published:** 2011-11-07

**Authors:** Stefania Bertolazzi, Francesco Lanzarotto, Barbara Zanini, Chiara Ricci, Vincenzo Villanacci, Alberto Lanzini

**Affiliations:** 1 Gastroenterology Unit: Spedali Civili and University. Piazzale Spedali Civili 1. 25123 Brescia. Italy; 2 Histopathology: Spedali Civili and University. Piazzale Spedali Civili 1. 25123 Brescia. Italy

## Abstract

**Background:**

Intestinal mucosa is leaky in celiac disease (CD), and this alteration may involve changes in hydrophobicity of the mucus surface barrier in addition to alteration of the epithelial barrier. The aims of our study were i) to compare duodenal hydrophobicity as an index of mucus barrier integrity in CD patients studied before (n = 38) and during gluten- free diet (GFD, n = 68), and in control subjects (n = 90), and ii) to check for regional differences of hydrophobicity in the gastro-intestinal tract.

**Methods:**

Hydrophobicity was assessed by measurement of contact angle (CA) (Rame Hart 100/10 goniometer) generated by a drop of water placed on intestinal mucosal biopsies.

**Results:**

CA (mean ± SD) of distal duodenum was significantly lower in CD patients (56° ± 10°)) than in control subjects (69° ± 9°, p < 0.0001), and persisted abnormal in patients studied during gluten free diet (56° ± 9°; p < 0.005). CA was significantly higher (62° ± 9°) in histologically normal duodenal biopsies than in biopsies with Marsh 1-2 (58° ± 10°; p < 0.02) and Marsh 3 lesions (57° ± 10°; p < 0.02) in pooled results of all patients and controls studied. The order of hydrofobicity along the gastrointestinal tract in control subjects follows the pattern: gastric antrum > corpus > rectum > duodenum > oesophagus > ileum.

**Conclusions:**

We conclude that the hydrophobicity of duodenal mucous layer is reduced in CD patients, and that the resulting decreased capacity to repel luminal contents may contribute to the increased intestinal permeability of CD. This alteration mirrors the severity of the mucosal lesions and is not completely reverted by gluten-free diet. Intestinal hydrophobicity exhibits regional differences in the human intestinal tract.

## Background

The intestinal barrier is an important defence mechanism to prevent the spreading of bacteria and toxins of different origin from intestinal lumen to the systemic circulation [1]. It consists of the epithelial barrier, an anatomical barrier between the luminal content and the host, and of the pre-epithelial barrier, a functional barrier mainly constituted of a mucus layer and by other factors such as the trefoil peptides, defensins and secretory IgA. The importance of the mucus layer as a component of the pre-epithelial barrier is supported by a large body of evidence in humans and in the animal in studies showing a protective effect against injury in the stomach [2,3], and a capacity to prevent bacteria and luminal toxins to come into direct contact with intestinal epithelium [4].

The intestinal barrier is altered in several diseases including celiac disease (CD) [5] an autoimmune disease that develops in genetically predisposed subjects exposed to ingestion of wheat gliadin and of related prolamines of barley and rye. This alteration of the intestinal barrier of CD results in increased intestinal permeability [6], a phenomenon attributed to excess production of a local peptide, zonulin, leading to disassembly of the tight junction structure that in physiological conditions seals the barrier and limits the paracellular passage of macromolecules, including the toxic fractions of gliadin [7]. This pathogenetic mechanism and the subsequent sub-epithelial events leading to intraepithelial lymphocytic infiltration and to mucosal atrophy have been extensively studied [8], but limited information is available on events involving the pre-epithelial component of the intestinal barrier, namely the mucous layer.

One way of assessing the functional integrity of the mucus layer is by measuring its hydrophobicity, a surface bio-physical property that affects adhesion of macromolecules, bacteria and toxins to the intestinal epithelia [9,10]. The importance of this characteristic in contributing to the integrity of the intestinal barrier is supported by the finding that decreased hydrophobicity has been documented in a variety of conditions including peptic ulcer disease [11], Helicobacter pylori infection and gastritis [12], and to accompany damage to the stomach or colon caused by non steroidal anti-inflammatory drugs [13], 2,4,6-trinitrobenzenesulphoric acid [14], LPS [15] or dextran sodium sulphate [16].

Mucosal hydrophobicity can be assessed by an empirical thermodinamic approach involving measurement of the contact angle subtended at the triple point where the solid-liquid, liquid-air and solid air interfaces meet following application of a drop of fluid to a solid surface [2]. The principle behind this technique is that a drop of fluid tend to form beads when applied on hydrophobic surfaces forming high contact angles at the triple point interface, and to spread on hydrophilic surfaces forming a small contact angle. Thus the higher is the contact angle the more hydrophobic is the surface.

The aim of our study was to assess the hyfrophobicity of the mucus barrier by measuring contact angle of gastro-intestinal mucosa exposed to a drop of saline in CD patients at diagnosis, and to assess the effect of gluten free diet (GFD). We also studied control subjects to obtain reference values of surface hydrophobicity of different regions of the gastro-intestinal tract in healthy humans. Preliminary validation studies were carried out to assess optimal experimental conditions and reproducibility.

## Methods

We collected mucosal biopsies at endoscopy in 3 different groups of patients consecutively recruited from December 2004 to January 2007. The first group consisted of control subjects referred to our Gastroenterology Unit for clinical and endoscopic evaluation. In all cases no abnormality was detected at endoscopy or histology except for minimal changes, and in all cases the final diagnosis was of functional disease. The second group consisted of CD patients diagnosed on the basis of positive anti- t-transglutaminases and/or antiendomysial antibodies and of a characteristic duodenal lesion. The third group consisted of CD patients on GFD for at least 1 year undergoing follow-up endoscopy, a standard procedure in our CD Clinic [17]. In these latter group adherence to GFD was assessed by interview using a 4 point Likert scale as previously described [18].

Following endoscopic examination biopsy specimens were taken 1 cm above the Z line in the oesophagous, 1 cm above the lesser curve and within 2 cm of the pylorus in the stomach, and at midway along the descending duodenum. Biopsies in the rectum were collected at 10 cm from the anal verge, and in the terminal ileum at 5 cm from the ileo-cecal valve. Biopsies were placed mucosal up on a piece of cellulose disk, and 1 unfixed biopsy specimen from each region was used for measurement of mucosal hydrophobicity by goniometry. Two more biopsies (4 in the case of duodenal biopsies) were fixed with formalin for subsequent histological examination.

Sections for histological examination were stained with H&E and presence and severity of inflammation was graded according to standard criteria. Duodenal biopsies have been graded according to modified Marsh classification [19,20], and immnuohistochemistry has been used to identify and count CD3+ intraepithelial lymphocytes.

For goniometry, freshly collected biopsy specimens oriented mucosal up were immediately washed with saline and placed on the stage of the goniometer (Rame/Hart 100/00 NJ, USA) fitted with a monochromatic light source and micrometer-activated syringe (Rame-Hart 100-10) for applying 5 μL of 0.15 M saline to the biopsy surface [21]. On application of the water drop two cross-hairs fitted within the microscope of the goniometer were aligned to the tangent of the air-saline drop-biopsy interface and the contact angle read off a scale incorporated in the eyepiece of the goniometer. All goniometric measurements have been carried out by one of us (SB) unaware of the site of biopsies and of endoscopic findings. The value of each measurement was the mean of 3 contact angle readings.

### Validation studies

The effect of drying of mucosal biopsies on CA measurements was assessed by allowing biopsies to air dry at room temperature after gentle washing, and by measuring contact angle at 10 min intervals starting from 10 minutes after collection. Reproducibility of CA measurements by the same goniometrist was assessed by calculating coefficient of variation of multiple measurements on biopsy specimens.

### Statistics

Results are expressed as means ± SD and as upper and lower 95% Confidence Interval of the mean. Unpaired t test was carried out for comparisons, and a p value < 0.05 was taken to indicate statistical significance for differences.

The study has been carried out in compliance with the Helsinki Declaration following approval by our Institutional Ethics Committee, and patients have signed an informed consent to the study.

## Results

Thirty-eight newly diagnosed CD patients on gluten containing diet, 68 CD patients on GFD and 105 controls (69 for upper and 36 for both upper and lower gastrointestinal tract studies) participated to the study. Anthropometric, serological and histological characteristics of patients and controls are summarized in table 1. All CD patients studied on gluten containing diet tested positive at CD related serology, and duodenal biopsy showed villous atrophy in 33 and lymphocytic duodenosis in 5. All patients studied on GFD given for 29 ± 10 months (range 12-35 months) were strictly adherent to GFD as assessed by interview, and all tested negative at CD related serology. Duodenal histology demonstrated persistent lymphocytic duodenosis in 60 patients, and mild villous atrophy in 5. No one of the control subjects tested positive at anti- t-transglutaminases antibodies serological screening.

**Table 1:** anthropometric and clinical characteristics of celiac patients studied at baseline, of patients studied during gluten free diet and of control subjects

Upper GI tract		Celiacs	Controls
	Baseline	GFD	
**n**	38	68	69
**F/M**	27/11	49/19	50/19
**age (years)**	34.5 ± 4.7	36.2 ± 2.2	48.9 ± 16.4*
**Marsh**			
**0**	0	2	57
**1**	0	0	3
**2**	5	60	8
**3a**	5	5	0
**3b**	5	1	0
**3c**	23	0	0
**Antibodies (n)**			
**tTg**	17	0	0
**EMA**	13	0	0
**t-TG + EMA**	8	0	0
**HP (n)**			
**positive**	8	10	13
**negative**	30	58	56
**Upper and lower GI tract**			**Controls**
**n**			36
**F/M**			21/15
**age (years)**			51.6 ± 20.5*

Biopsies of the lower gastrointestinal tract have been obtained in control subjects only. GFD = gluten free diet GI = gastrointestinal. * = p < 0.0001

### Validation studies (figure 1)

The effect of mucosal drying on goniometric measurements was studied in 15 gastric, 12 duodenal and 12 rectal biopsies. Contact angle increased in gastric biopsies from 59° ± 4° at time 10 minutes to 72° ± 4° at time 20 minutes and remained stable thereafter at time 30 (68° ± 3°), 40 (72° ± 4°), 50 (70° ± 3°) and 60 minutes (67° ± 3°). Similar pattern with early increase in value and a plateau effect after 20 minutes was observed for biopsies taken in the duodenum (53° ± 2°, 64° ± 3°, 65° ± 2°, 65° ± 2°, 61° ± 2° and 61° ± 2° at time 10, 20, 30, 40 50 and 60 minutes respectively) and in the rectum (57° ± 3°, 69° ± 3°, 70° ± 2°, 71° ± 4°, 69° ± 3° and 69° ± 4° at time 10, 20, 30, 40 50 and 60 minutes respectively). Coefficient of variation for 5 CA readings in 6 biopsy specimens of gastric corpus was 8%, 3%, 5%, 2%,2% and 5%, respectively.

Based on these validation results all subsequent measurements of contact angle were carried on mucosal biopsies within 20-60 min from collection following gentle biopsy washing with saline.

**Figure 1:**
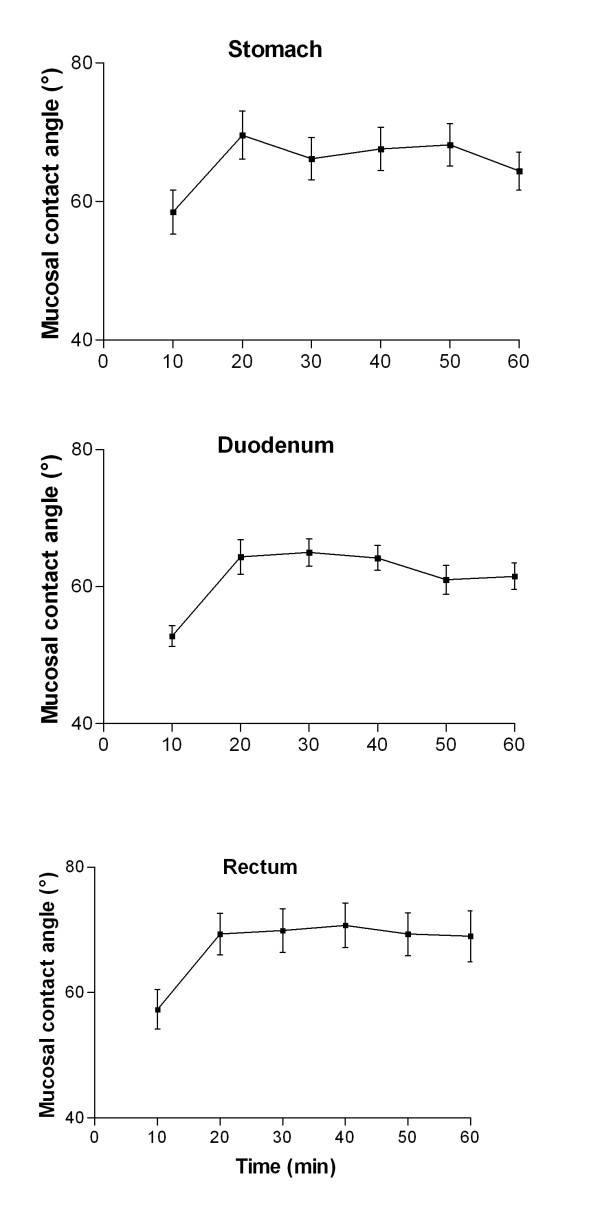
**Validation study: effect of mucosal drying on gastric, duodenal and rectal mucosal contact angle**.

### Surface hydrophobicity in celiac disease and effect of gluten free diet (figures 2, 3)

**Figure 2:**
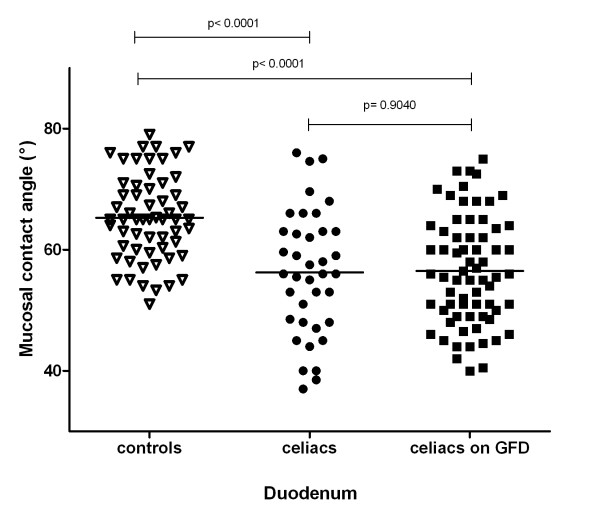
**Contact angle of duodenal mucosa in control subjects, celiac patients and celiac patients on gluten-free-diet GFD = gluten free diet**.

**Figure 3:**
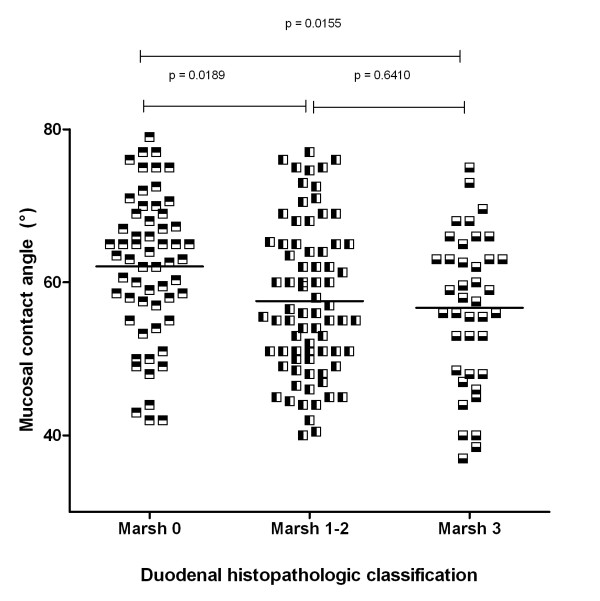
**Contact angle of duodenal mucosa plotted according to histopathologic classification (Marsh grade **[19]) **of duodenal specimens in all patients and controls studied**.

Contact angle of duodenal biopsies (Figure 2) was significantly lower in CD patients (56° ± 10°: 95% CI: 53°-60°) than in healthy subjects (65° ± 7°: 95% CI: 63°-67°: p < 0.0001), and remained virtually unchanged in CD patients during GFD 56° ± 9° (95% CI: 54°-59°; p = 0.904). Contact angle of duodenal mucosa was not affected by Helicobacter pylori infection in CD patients (57° ± 10° vs 52° ± 11°), CD patients on GFD (57° ± 9° vs 55° ± 9°) and controls (61° ± 10° vs 65° ± 10° for Helicobacter pylori negative and positive, respectively) and was not related to gender or age in all 3 groups (Pearsons' r = 0.02781 for CD, r = 0.1157 for CD on GFD and r = 0.0883 for controls).

By contrast with results in duodenal biopsies, there was no difference in contact angle measurements on antral biopsies between CD patients (69° ± 9°; 95% CI: 65°-72°), CD patients during GFD (68° ± 10°; 95% CI: 66°-71°) and control subjects 72° ± 8° (95% CI: 70°-73°), and there was also no difference on biopsies taken from gastric corpus the 3 groups (70° ± 9°; 95% CI: 68°-71°//67° ± 10°; 95% CI: 63°-71°//67° ± 10°; 95% CI: 65°-70°, respectively).

Figure 3 shows results of contact angle measured on duodenal mucosa plotted independently of clinical diagnosis as a function of histopathologic characteristics of the mucosa in all 175 patients in the 3 groups involved in the study. Contact angle was higher in mucosal biopsies classified as Marsh 0 (62° ± 9°; 95% CI: 60°-64°: p < 0.0001) than in those classified as Marsh 1-2 (58° ± 10°; 95% CI: 55°-60°: p = 0.0189) and as Marsh 3 (57° ± 10°; 95% CI: 54°-60°: p = 0.0155).

### Regional differences in surface hydrophobicity (figures 4 and 5)

**Figure 4:**
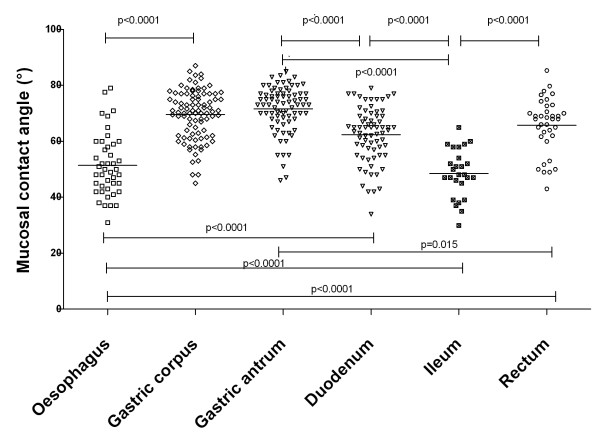
**Mucosal contact angle at different locations in the gastro-intestinal tract of control subjects**.

Regional differences for CA measurement were present along the gastro-intestinal tract in control subjects (Figure 4). CA was 51° ± 11° (95% CI: 45° -50°) in 67 oesophageal, 70° ± 9° (95% CI: 68° -71°) in 90 gastric body, 72° ± 8° (95% CI: 70°- 73°) in 78 gastric antrum, 62° ± 10° (95% CI: 60°- 65°) in 69 duodenal, 49° ± 9° (95% CI 45° -52°) in 26 distal ileum and 66° ± 10° (95% CI: 63° -69°) in 36 rectal biopsies.

**Figure 5:**
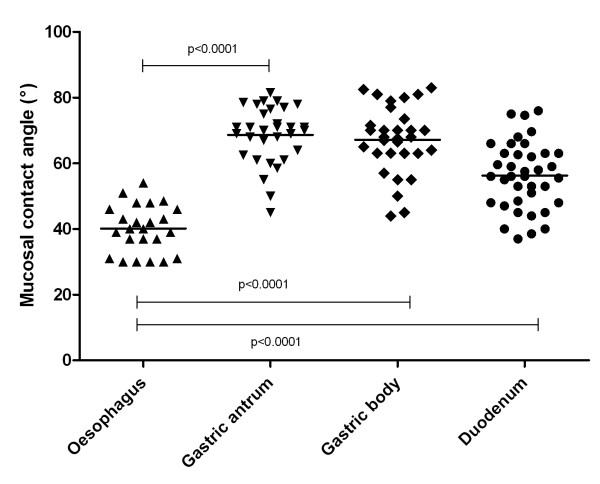
**Mucosal contact angle at different locations in the gastro-intestinal tract of celiac patients**.

Regional differences for CA measurement were maintained in CD patients in studies limited to the upper gastro-intestinal tract (Figure 5). CA was lower on oesophageal (40° ± 7° (95% CI: 37°-43°) than on gastric antral biopsies (69° ± 9° (95% CI: 65°-72°). CA of duodenal biopsies (56° ± 10° (95% CI: 53°-60°) was lower than that of gastric antral 69° ± 9° (95% CI: 65°-72°: p < 0.0001) and corpal biopsies 56° ± 10° (95% CI: 53°-60°: p < 0.0001). Similar results have been obtained in CD patients during GFD with lower values for contact angle in duodenal 56° ± 9° (95% CI: 54°-59°) than in gastric antral 69° ± 10° (95% CI: 66°-71°: p < 0.0001 and corpal biopsies 67° ± 10° (95% CI: 65°-70°: p < 0.0001).

## Discussion

In the present study we assessed surface hydrophobicity of gastrointestinal mucosa as an index of the functional integrity of the mucus layer, a layer that acts as a "closing seal"[22] of the intestinal barrier. This bio-physical characteristic can be studied by measuring contact angles formed by sessile water droplets placed on freshly collected biopsies. The interrelation between contact angle and surface energy assumes that the surface on which contact angle is measured is smooth and homogenous [23], and this is obviously not the case for mucosal biopsies. Furthermore, drying, presence of debris and trauma of biopsy collection may cause non-physiological changes. Although these observations are a matter of concern, drying has been reported by Hills [24] to provide a more conservative estimate of the energy reduction that would occur if the mucus remained in the physiological hydrated state. Our validation study confirms that contact angle increases during mucosal drying to a point when it remains stable and reproducible up to at least 60 min following biopsy collection.

The main aspect of our study is that we assessed the hydrophobicity of the duodenal mucus barrier in CD patients studied at diagnosis and in CD patients on GFD. This assessment was prompted by a large body of evidence indicating that intestinal permeability to macromolecules is increased in CD patients [6,25,26]. This phenomenon has been extensively studied in relation to the epithelial component of the intestinal barrier, and shown to involve altered regulation of the tight junctions that provide contiguity of epithelial cells and regulate the traffic trough the paracellular pathway. Zonulin has been identified as a protein with capacity to disassemble TJ [7] as an early event in the development of CD [27] favouring the passage of gliadin toxic fragments via the paracellular pathway from the intestinal lumen to the submucosa. Although the validity of this model has been questioned [28], the pathogenetic role of increased permeability in development of CD is firmly established.

Our study clearly indicates that the mucus barrier is altered in CD, and that this defect is not restored to normal during GFD as indicated by the observation that hdyrophobicity of duodenal mucosa is lower in CD patients than in control subjects, and that this abnormality persists during GFD. These differences cannot be accounted by artefacts due to Helicobacter pylori infection and by differences in age, as reported by others for gastric biopsies [29], because there was no relationship in our patients and in controls between these parameters and duodenal CA. Furthermore, the younger age of our CD compared with controls should point, if anything, to a higher CA, an opposite effect of what we have found.

The reduced hydrophobicity is specific for duodenal mucosa in CD, as indicated by the finding that hydrophobicity of gastric mucosa was similar in CD patients and in controls.

The mechanism involved in this bio-physical alteration of the duodenal mucus barrier is unclear. Previous studies have reported alteration of the structure of duodenal mucus in CD patients associated with defect of the trefoil factors family [30] and with the structure and secretory pattern of mucus glycoprotein [31], defects that may affect stability of mucus. We could not further investigate the mechanism involved in reduced hydrophobicity of duodenal mucus barrier, but it is clear from our results a relationship with the anatomical changes of the mucosa, independently of clinical diagnosis. This is suggested by our finding that by plotting together results of duodenal CA obtained in control subjects with those obtained in CD patients off and on GFD, there was a clear-cut dependency of reduced mucus hydrophobicity on severity of duodenal histopathology (Figure 3). This was irrespective of clinical diagnosis because the group with normal histology comprised CD patients on GFD in addition to control subjects, and the group with Marsh-1-2 lesion comprised normal subjects in addition to CD patient on or off GFD. On the basis of our results we cannot firmly establish the nature of this relationship, and although a cause-effect mechanism cannot be proven it seems to us plausible because of the independence of the aetiology of mucosal damage.

To what extent the alteration in mucus barrier contributes to the pathogenesis of CD, and whether this alteration is a primary defect is totally unclear but it fits a paradigm for initiation of autoimmune diseases proposed by Arrieta et al [5]. According to this model, undigested gliadin fractions are kept separate from sub-epithelial immune system by a competent intestinal barrier. Disease activation occurs when factors with capacity of altering the intestinal barrier integrity favour paracellular transit of toxic fractions of gliadin, and these initiating factors include drugs such as NSAIDs or infections that are all well known factors for their capacity to disrupt the mucus barrier [13,32,33]. The late onset of CD in genetically predisposed subjects fits with this hypothesis on the role of initiating factors.

Another aspect of our study is that we looked at regional differences of hydrophobicity of intestinal mucosa in controls and in CD, and found marked differences in different intestinal regions. These differences are qualitatively similar to those reported by Spychal et al [21] in humans showing high hydrophobicity in gastric and in rectal mucosa, with lower values in the duodenum and in the ileum. This regional difference is common to many mammalian species [10,16,21,34,35] although slight species-related differences occur. By contrast with results in the animal, in our study mucosal hydrophobicity was lower in the distal ileum than in the duodenum. No other study to our knowledge has reported results for hydrophobicity of distal ileum in humans, but the tendency for hydrophobicity to be reduced from proximal to distal duodenum reported by Spychal [21] in healthy subjects is consistent with our results. The low hydrophobicity in the oesophagus observed in our study is also novel, and explains the susceptibility of oesophageal mucosa to refluxed gastric acid because hydrophobicity is important in repelling the diffusion of hydrogenions [2] from coming in contact with the epithelial cells.

## Conclusions

In conclusion our study has provided evidence that the mucus barrier, an important component of the pre-epithelial barrier, is altered in CD patients. We speculate that the resulting decreased capacity of the mucus barrier to repel luminal contents because of low hydrophobicity and to act as a "closing seal"[22] may potentially contribute to the increased intestinal permeability characteristic of CD. Although the model of intestinal hydrophobicity as a "closing seal" has as yet to be proven, it has been proven that this bio-physical property represents a valid criterion for assessing the protective function of the mucus layer. Studies in the animal and pilot studies in humans suggest that mucus layer hydrophobicity can be increased by oral administration of phospholipids [36], and this may prove of value as adjuvant treatment in CD patients with incomplete response to GFD. Our study has also confirmed regional differences in surface hydrophobicity of the mucus layer of control subjects, and has provided reference values that may be of value for further comparative studies in disease conditions other than CD.

## Competing interests

The authors declare that they have no competing interests.

## Authors' contributions

SB carried out all goniometric measurements and contributed to planning the study, and to analysis and expression of results

FL carried out all endoscopies, provided all bioptic material for the study and contributed to planning of the study.

BZ contributed to identification and selection of the patients and controls, and contributed to analysis and expression of results

CR contributed to identification and selection of the patients and controls, and contributed to analysis and expression of results

VV examined and classified all endoscopic biopsies.

AL planned the study, contributed to analysis and expression of results and wrote and edited the manuscript.

All authors read and approved the final manuscript

## Pre-publication history

The pre-publication history for this paper can be accessed here:

http://www.biomedcentral.com/1471-230X/11/119/prepub
